# Clinical efficacy, postoperative complication risks, and parental satisfaction in pediatric patients receiving tension-reducing suture treatment for facial lacerations

**DOI:** 10.3389/fped.2025.1633189

**Published:** 2025-10-13

**Authors:** Zijing Mi, Pingying Zeng, Weiyuan Yang, Haiyang Sun, Ping Yao

**Affiliations:** Department of Plastic Surgery, Hangzhou Plastic Surgery Hospital, Hangzhou, Zhejiang, China

**Keywords:** facial laceration, Children, tension-reducing suture, clinical efficacy, Vancouver Scar Scale (VSS)

## Abstract

**Objective:**

To evaluate the clinical efficacy, postoperative complication risks, and parental satisfaction of tension-reducing sutures in pediatric patients with facial lacerations.

**Methods:**

A retrospective cohort analysis was conducted on 122 pediatric patients (aged 1–12 years) with facial lacerations who met predefined inclusion criteria (e.g., aged 1–12 years, wound length 1–5 cm, etc.; see Methods for details) and underwent surgical treatment at our hospital between January 2020 and August 2024. Based on the surgical technique received, the patients were divided into the tension-reducing suture group *n* = 61) and the conventional suture group (*n* = 61). The two groups were compared regarding baseline clinical characteristics, clinical efficacy, scar formation, surgical features, postoperative complications, and parental satisfaction.

**Results:**

The primary healing rate (Grade A) in the tension-reducing suture group was 88.5% (54/61), significantly higher than that in the conventional suture group (73.8%, 45/61), with a statistically significant difference (*χ*^2^ = 4.340, *p* = 0.037). At postoperative 1 month [(4.25 ± 1.16) vs. (4.80 ± 1.21)] and 3 months [(3.69 ± 1.03) vs. (4.08 ± 1.10)], the Vancouver Scar Scale (VSS) scores in the tension-reducing suture group were significantly lower than those in the conventional suture group (*t* = −2.594, −2.044; *p* = 0.011, 0.043). The tension-reducing suture group had longer operative time [(55.08 ± 11.23) min vs. (50.16 ± 10.46) min], more suture layers [(2.85 ± 0.54) vs. (2.61 ± 0.58)], and more sutures [(41.48 ± 8.42) vs. (38.49 ± 6.20)] compared to the conventional suture group (t = 2.502, 2.406, 2.229; *p* = 0.014, 0.018, 0.028). No significant difference was observed in intraoperative blood loss between the two groups (*p* > 0.05). The postoperative complication rate was 4.9% (3/61) in the tension-reducing suture group and 14.8% (9/61) in the conventional suture group, with no statistically significant difference (*χ*^2^ = 3.327, *p* = 0.068). Parental overall satisfaction was 93.4% (57/61) in the tension-reducing suture group and 80.3% (49/61) in the conventional suture group, showing a statistically significant difference (*χ*^2^ = 4.604, *p* = 0.032).

**Conclusion:**

Tension-reducing suture technique is more conducive to promoting primary wound healing and reducing scar formation in pediatric patients. Additionally, this suturing method was associated with a trend towards fewer complications while improving parental satisfaction with surgical outcomes.

## Introduction

Due to their weak self-protection ability, high activity levels, and frequent exposure of the face, children exhibit a consistently high incidence of head and facial lacerations. According to clinical data, facial lacerations account for 20%–30% of all pediatric traumas, with children aged 1–12 being the most affected group (1). As a critical functional and aesthetic organ, the face not only performs essential functions such as emotional expression, respiration, and mastication, but its structural integrity also plays a vital role in social interaction and self-perception. Children have relatively thin facial skin, loose subcutaneous tissue, and abundant blood supply, which promotes rapid wound healing. However, frequent facial muscle movements mean that improper suturing can easily lead to poor wound alignment due to persistent tension, resulting in noticeable scarring (2). Furthermore, as children are in a stage of growth and development, scars may stretch and deform along with the growth of bones and soft tissues, exacerbating aesthetic and functional impairments. These visible differences can become a focus of peer attention once children reach school age, potentially leading to teasing or social isolation, thereby negatively affecting psychological development (3). Therefore, selecting the best suturing method for pediatric facial lacerations is a core clinical issue aimed at reducing scar formation and preserving both function and appearance. This approach is directly related to the child's short-term recovery, psychological well-being, and long-term quality of life.

As an important surgical suturing technique, tension-reducing sutures effectively promote wound healing and reduce scar formation by alleviating the tension on wound edges during suturing. This technique plays a pivotal role in the surgical treatment of various types of facial lacerations (4–6). Compared to conventional suturing methods, tension-reducing sutures provide better approximation of wound edges, reduce wound tension, decrease the likelihood of hypertrophic scarring, and improve aesthetic outcomes after wound healing (7). Previous studies comparing the application of conventional suturing and tension-reducing sutures in patients with facial lacerations have found that the tension-reducing suture group experienced shorter wound healing time and significantly less scarring (8). Other studies have also reported that reduced facial scarring following tension-reducing suture treatment led to a notable alleviation of psychological distress in patients (9). Although there is existing research on the use of tension-reducing sutures in the treatment of facial lacerations, most studies have focused on adult populations, with limited dedicated research on pediatric groups. Moreover, the impact of children's physiological characteristics—such as thinner dermal layers and looser collagen fiber arrangement—on suturing outcomes has not been sufficiently considered. Therefore, this study aims to evaluate the effects of tension-reducing sutures vs. conventional suturing in the treatment of facial lacerations in children. The findings are expected to provide a theoretical basis for selecting suturing methods and guiding surgical decision-making in pediatric facial laceration cases, as well as to contribute clinical evidence for further enhancing the standardization and humanization of pediatric facial trauma treatment.

## Methods

### Study participants

Pediatric patients with facial lacerations who underwent surgical treatment at Hangzhou Plastic Surgery Hospital between January 2020 and August 2024. Patients were divided into two groups based on the surgical technique they actually received: the tension-reducing suture group (*n* = 61) and the conventional suture group (*n* = 61).

The inclusion criteria were as follows: aged 1–12 years; acute facial lacerations with an injury-to-treatment interval within 24 h; and a wound length between 1 and 5 cm. The exclusion criteria were defined as the presence of concurrent severe organ injuries or systemic diseases; chronic wounds or infected facial lacerations; a history of allergy to suture materials; or any other contraindications for surgical intervention.

### Treatment description

Conventional suture group: The pediatric patient was treated using the conventional suturing method. First, wound debridement was performed by repeatedly irrigating the wound and surrounding skin with normal saline to avoid secondary trauma. The wound and surrounding skin were then disinfected with povidone-iodine, ensuring the disinfection area extended at least 5 cm beyond the wound edges. Under local infiltration anesthesia, a scalpel was used to trim the wound edges, removing necrotic and severely contused tissue to create clean and even wound margins. Hemostasis was achieved using electrocautery or silk ligation for bleeding points within the wound. For suturing, 4-0 silk suture was selected for full-thickness interrupted sutures. The needle was inserted perpendicular to the skin surface, ensuring the depth reached the base of the wound. The stitch spacing was maintained at 3–5 mm, with a margin distance of 2–3 mm. Each suture was individually tied to prevent tension between stitches, which could adversely affect wound healing. During suturing, care was taken to ensure precise and even approximation of the wound edges, avoiding inversion or eversion of the skin. The postoperative care protocol is as follows: ① Wound care: Change the dressing on postoperative day 1 and observe for wound bleeding and redness. For the first 3 days, clean the skin around the wound daily with sterile saline solution, avoiding contact with water. After 3 days, gently wipe with clean water; rubbing is strictly prohibited. ② Antibiotic use: For clean wounds, administer oral amoxicillin and clavulanate potassium dry suspension (20–40 mg/kg body weight, twice daily) for 3 days. For contaminated wounds (e.g., injuries from falls or collisions), administer intravenous cefturoxime sodium (30–50 mg/kg body weight, twice daily) for 5 days. ③ Activity restrictions: Avoid strenuous exercise for 1 week post-surgery and reduce excessive facial muscle movement to prevent increased wound tension.

**Figure 1:**
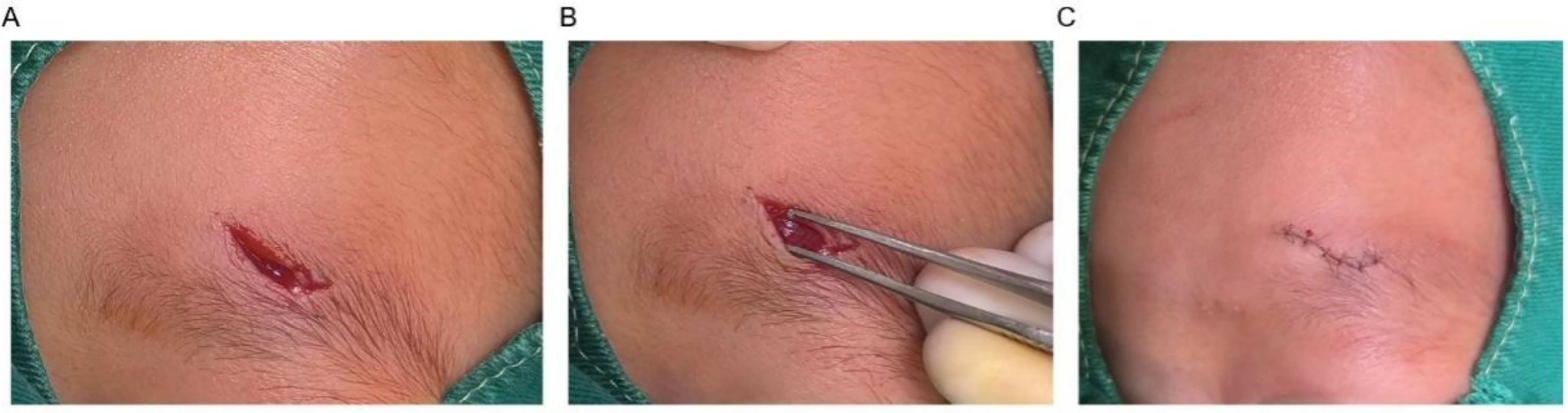
A pediatric patient with a facial laceration treated with the tension-reducing suture technique. **(A)** Preoperative view; **(B)** Intraoperative view demonstrating wound depth; **(C)** postoperative view.

Tension-reducing suture group: The pediatric patient was treated with tension-reducing sutures. The procedures for wound debridement, disinfection, and hemostasis were the same as those in the conventional suture group. A layered suture technique was employed during closure. First, the subcutaneous tissue was sutured using 5-0 absorbable sutures [VICRYL Plus (Polyglactin 910) sutures, Johnson & Johnson Medical (Suzhou) Ltd., China)], ensuring that the knots were buried within the deeper tissue. The needle depth was adjusted to achieve proper approximation of the subcutaneous tissue, with a stitch spacing of 4–6 mm. A specialized dermal anchoring tension-reduction technique was applied to the dermis using 6-0 absorbable sutures in an interrupted fashion. The entry point was approximately 2–3 mm from the wound edge, while the exit point was placed in the deeper dermal layer. The suture was passed through the deep tissue and then brought out through the opposite side at a more superficial dermal level before being tied on the wound surface. For the epidermal closure, a continuous intradermal suture was performed using 7-0 cosmetic sutures. The needle was inserted from one end of the wound with a stitch spacing of 1–2 mm, and the suture was continuously passed through the dermis before being tied off at the opposite end of the wound. The postoperative care protocol was consistent with that of the conventional suture group. An example is shown in Figure 1. All procedures were performed by the same surgical team, consisting of three attending physicians with over five years of experience in pediatric plastic surgery and one associate chief physician. Preoperative standardized training was conducted to ensure uniform operative procedures, the same anesthesia protocol was applied during surgery, and dedicated personnel recorded surgical parameters to minimize technical variations among different operators.

### Analyzed variables

(1) General clinical data and disease characteristics: the age, gender, cause of injury, wound length, depth, and other information of children in both groups were recorded and compared in detail. The causes of injury were mainly categorized into falls, impact injuries, sharp object lacerations. Wound length was measured using a ruler, with precision to the millimeter. Wound depth was assessed via probe detection and measured in millimeters, and then classified into three levels: superficial, moderate, and deep. Among these, superficial wounds involved only the epidermal layer, moderate wounds extended to the dermal layer, and deep wounds reached the subcutaneous tissue or even deeper structures. (2) Clinical Efficacy Evaluation: Wound healing status was assessed according to the Incision Healing Grading Criteria, categorized as Grade A healing, Grade B healing, and Grade C healing (10). Grade A healing refers to satisfactory wound healing without adverse reactions such as redness, swelling, exudation, or pain, resulting in minimal scarring that does not affect facial aesthetics. Grade B healing indicates mild inflammatory reactions at the wound site, such as slight redness, swelling, or minimal exudation without suppuration, with relatively noticeable scarring that has some impact on aesthetics. Grade C healing involves wound suppuration requiring interventions like incision and drainage, resulting in significant scarring that severely affects facial aesthetics. The Grade A healing rate was calculated as (Number of Grade A healing cases/Total number of cases) × 100%, providing a direct reflection of the excellent/good rate of wound healing. (3) Scar Condition (11): The Vancouver Scar Scale (VSS) was used for evaluation, which assesses scars based on four parameters: pigmentation, height, vascularity, and pliability. Each parameter is assigned a score within a specific range. Pigmentation is categorized as normal (0 points), hypopigmented (1 point), mixed (2 points), or hyperpigmented (3 points); height as normal (0 points), <2 mm (1 point), 2–5 mm (2 points), or >5 mm (3 points); vascularity as normal (0 points), pink (1 point), red (2 points), or purple (3 points); and pliability as normal (0 points), supple (1 point), firm (2 points), banding (3 points), contracture (4 points), or unyielding (5 points). The total score is the sum of all parameter scores, ranging from 0 to 15, with higher scores indicating more severe scarring. VSS assessments were independently conducted by two attending plastic surgeons who were not involved in the surgeries. The intraclass correlation coefficient (ICC) indicated excellent inter-rater reliability [ICC = 0.89 (95% CI: 0.82–0.94)]. (4) Surgical Characteristics: The intraoperative characteristics of both groups were recorded, including operative time, blood loss, number of suture layers, and number of stitches. The operative time was measured from the initiation of anesthesia to the completion of suturing. Blood loss was estimated by the volume collected in the suction device and the amount absorbed by surgical gauze during the procedure. The number of suture layers was documented based on the actual surgical procedure, specifically distinguishing between subcutaneous tissue sutures and skin sutures. The number of stitches was counted according to the actual sutures performed. (5) Postoperative Complications: The occurrence of complications within one month after surgery was closely observed in both groups, primarily including wound hemorrhage, infection, dehiscence. (6) Parental Satisfaction Survey: A self-designed parental satisfaction questionnaire was administered to the parents one month after the surgery. Satisfaction was categorized into three levels: “very satisfied”, “satisfied”, and “dissatisfied”. “Very satisfied” indicated that parents were highly satisfied with all aspects without any complaints; “satisfied” meant parents were generally content; “dissatisfied” indicated that parents had significant concerns regarding certain aspects. Parental satisfaction was calculated as: (Number of “very satisfied” + Number of “satisfied”)/Total number of cases × 100%.

### Statistical methods

Statistical analysis was performed using SPSS® 26.0. Measurement data with a normal distribution were expressed as mean ± standard deviation (mean ± SD), and comparisons between independent samples were made using a *t*-test. Ranked or count data were presented as cases or percentages, with intergroup comparisons performed using the chi-square test (*χ*^2^). A *p*-value <0.05 was considered statistically significant.

### Sample size and power

As this was a retrospective study that included all eligible patients during the investigation period, a prospective sample-size calculation was not performed. A *post-hoc* power analysis was conducted using G*Power® 3.1.9.7 software. For the primary outcome of overall complication rate (14.8% in control vs. 4.9% in tension-reducing suture group), with a total sample size of 122, an alpha of 0.05, and an effect size (*w*) of 0.20, the achieved power was 55.2%.

### Ethical considerations

This study was conducted in accordance with the Declaration of Helsinki and was approved by the Institutional Ethics Committee of Hangzhou Plastic Surgery Hospital (Approval No. HZLL20200005). Written informed consent was obtained from the parents or legal guardians of all participating children for both the surgical procedure and the publication of any potentially identifiable data, including clinical photographs. In all presented photographs, all identifying features (e.g., eyes) have been masked to ensure patient anonymity.

## Results

A total of 122 pediatric patients with facial lacerations who underwent surgical treatment were included. Among them, there were 68 males (*n* = 68, 55.7%) and 54 females (*n* = 54, 44.3%), with an age range of 4–11 years and a mean age of 7.66 (1.46) years. The causes of injury were falls (*n* = 52, 42.6%), collisions (*n* = 42, 34.4%), and sharp object lacerations (*n* = 28, 23.0%). The wound locations were distributed as follows: forehead (*n* = 41, 33.6%), eyebrow arch (*n* = 31, 25.4%), cheek (*n* = 27, 22.1%), and submental region (*n* = 23, 18.9%). The two groups were comparable at baseline, with no statistically significant differences in gender, age, cause of injury, injury site, wound length, or wound depth (all *p* > 0.05, Table 1).

**Table 1:** Comparison of general clinical data and disease characteristics between the Two groups [(mean ± SD), cases].

Group	Number of cases (*n*)	Gender (male/female) (*n*)	Age (years) (mean ± SD)	Cause of injury (fall/collision injury/sharp object laceration) (*n*)	Site of injury (forehead/superciliary arch/cheek/submental region) (*n*)	Wound length (cm) (mean ± SD)	Wound depth (superficial/moderate/deep) (*n*)
Tension-reducing suture group	61	33/28	7.77 ± 1.45	27/20/14	22/15/13/11	4.04 ± 0.83	18/32/11
Conventional suture group	61	35/26	7.54 ± 1.48	25/22/14	19/16/14/12	4.01 ± 0.62	19/30/12
*χ*^2^/t		0.133	0.865	0.172	0.332	0.199	0.135
*p*		0.715	0.389	0.918	0.954	0.843	0.935

The excellent healing rate (Grade A) in the tension-reducing suture group was 88.5% (54/61), which was significantly higher than that in the conventional suture group (73.8%, 45/61), with a statistically significant difference (*p* < 0.05). At 1 month and 3 months postoperatively, the VSS scores of the tension-reducing suture group were significantly lower than those of the conventional suture group (*p* < 0.05), as detailed in Table 2.

**Table 2:** Differences in clinical efficacy and scar formation between the Two groups of pediatric patients [$\left(\bar{x}\pm s\right)$, *n*].

Group	Number of cases	Grade A healing		VSS score (points)	
		Number of cases	Healing rate (%)	1 month postoperative	3 months postoperative
Tension-reducing suture group	61	54	88.5	4.25 ± 1.16	3.69 ± 1.03
Conventional suture group	61	45	73.8	4.80 ± 1.21	4.08 ± 1.10
*χ*^2^/*t*		4.340		−2.594	−2.044
*p*		0.037		0.011	0.043

The operative time, number of suture layers, and number of sutures in the tension-reducing suture group were significantly higher than those in the conventional suture group (*p* < 0.05). However, there was no statistically significant difference in intraoperative blood loss between the two groups (*p* > 0.05). Refer to Table 3 for details.

**Table 3:** Differences in surgical characteristics between the Two groups of pediatric patients $\left(\bar{x}\pm s\right)$.

Group	Number of cases	Operation time (min)	Blood loss (ml)	Suture layers (layers)	Suture stitches (stitches)
Tension-reducing suture group	61	55.08 ± 11.23	15.10 ± 4.44	2.85 ± 0.54	41.48 ± 8.42
Conventional suture group	61	50.16 ± 10.46	15.25 ± 3.75	2.61 ± 0.58	38.49 ± 6.20
*t*		2.502	−0.198	2.406	2.229
*p*		0.014	0.843	0.018	0.028

The postoperative complication rate in the tension-reducing suture group was 4.9% (3/61), including 1 case of bleeding, 1 case of wound dehiscence, and 1 case of hypertrophic scarring. In the conventional suture group, the postoperative complication rate was 14.8% (9/61), consisting of 3 cases of bleeding, 1 case of wound infection, 2 cases of dehiscence, and 4 cases of hypertrophic scarring. The difference in postoperative complication risks between the two groups was not statistically significant (*p* > 0.05). See Table 4 for details.

**Table 4:** Difference in postoperative complication risks between the Two groups [cases].

Group	Number of Cases	Operation time (min)	Blood loss (ml)	Suture layers (layers)	Suture stitches (stitches)	Number of Cases
Tension-reducing suture group	61	1 (1.6)	0	1 (1.6)	1 (1.6)	4.9
Conventional suture group	61	3 (4.9)	1 (1.6)	2 (3.4)	3 (4.9)	14.8
*χ* ^2^		0.258	0.000	0.000	0.258	3.327
*p*		0.611	1.000	1.000	0.611	0.068

The overall satisfaction rate among parents in the tension-reducing suture group was 93.4% (57/61), while that in the conventional suture group was 80.3% (49/61). Parental satisfaction in the tension-reducing suture group was significantly higher than in the conventional suture group (*p* < 0.05), as illustrated in Table 5.

**Table 5:** Difference in parental satisfaction between the two groups [cases].

Group	Number of cases	Very satisfied	Satisfied	Dissatisfied	Satisfaction rate (%)
Tension-reducing suture group	61	38 (62.3)	19 (31.1)	4 (6.6)	93.4
Conventional suture group	61	27 (44.3)	23 (37.7)	11 (18.0)	80.3
*χ* ^2^					4.604
*p*					0.032

## Discussion

The facial skin is relatively thin and richly supplied with blood vessels and nerves, providing a favorable nutritional foundation for wound healing. However, the frequent muscle activity and dynamic facial expressions increase the susceptibility of facial wounds to tension during the healing process (12). When a wound is subjected to tension, the edges are subjected to mechanical stress, which may impede proper healing (13). Previous studies have indicated that tension-reducing suture techniques, through layered suturing and specialized tension-relieving methods, is designed to effectively distribute wound tension (14). In this study, we observed a higher rate of primary healing in the tension-reducing suture group, likely attributable to the multi-layered suturing and tension-reducing approach, which allowed the wound edges to heal under low tension, promoting cellular proliferation and migration, thereby enhancing the primary healing rate. Consistent with our findings, some studies have suggested that tension-reducing sutures can minimize tissue damage at the wound edges, reduce inflammatory cell infiltration and the release of inflammatory cytokines, thereby mitigating the negative impact of inflammation on wound healing (15, 16). In this study, the application of tension-reducing sutures in the tension-reducing suture group may have facilitated scar formation in a more stable environment, reducing excessive scar hyperplasia and contracture, resulting in finer and flatter scars (17). Furthermore, our study found that the postoperative VSS scores in the tension-reducing suture group were significantly lower than those in the conventional suture group, suggesting that this suturing method may further promote early wound healing. By reducing wound tension, tension-reducing sutures enable closer approximation of wound edges, improving local blood circulation and nutrient supply, thereby accelerating the healing process and providing a strong foundation for the patient's early recovery (18).

In the treatment of pediatric facial lacerations, the occurrence of postoperative complications can significantly impair wound healing and patient recovery (19). This study found that tension-reducing suture techniques may potentially reduce the risk of complications such as postoperative wound bleeding, infection, dehiscence, and hypertrophic scarring in children. The face has abundant blood supply, making bleeding a common issue after injury. Tension-reducing sutures employ a layered closure approach, using 5-0 absorbable sutures for tight subcutaneous tissue approximation, which effectively ligates bleeding points, eliminates dead space, and reduces gaps within the wound, thereby decreasing the likelihood of bleeding (20). Compared to conventional suturing, tension-reducing sutures provide more precise tissue alignment, better vascular compression, and reduced blood oozing (21). Additionally, tension-reducing sutures minimize wound edge tension, ensuring tight approximation and lowering the risk of bacterial invasion (5). The close apposition reduces dead space within the wound and prevents bacterial colonization. Furthermore, specialized techniques such as dermal retention sutures for tension dispersion and continuous intradermal suturing redistribute tension to deeper tissues, alleviating surface skin tension and reducing the likelihood of wound dehiscence (22). Dermal retention sutures effectively disperse tension in the dermal layer, preventing skin surface separation due to excessive tension, while continuous intradermal suturing enhances wound stability by ensuring tight epidermal alignment (23). In conclusion, although no statistically significant difference in complication rates was observed between the two groups, a strong numerical trend favoring the tension-reducing suture group was noted (4.9% vs. 14.8%, *p* = 0.068). The clinical relevance of this observed reduction, in light of the study being underpowered to detect it, warrants further investigation in larger trials.

Parental satisfaction is one of the important indicators for evaluating the quality of medical services. In the treatment of pediatric facial lacerations, factors such as surgical outcomes, doctor-patient communication, and postoperative guidance play a critical role in parental satisfaction, where tension-reducing sutures have shown a positive facilitating effect (24, 25). This study found that the satisfaction rate among parents in the tension-reducing suture group reached 93.4%, significantly higher than the 80.3% in the conventional suture group, indicating that the application of tension-reducing sutures was associated with significantly higher parental satisfaction in the treatment of facial lacerations. As the most prominent part of the body, the aesthetic appearance of the face is crucial to a child's life and psychological development. Tension-reducing sutures can effectively improve the primary healing rate of wounds and reduce scar formation, which is key to enhancing parental satisfaction. The favorable surgical outcomes in the tension-reducing suture group resulted in excellent facial recovery for the children, meeting parents' expectations regarding their child's facial aesthetics, thereby increasing their satisfaction with the treatment. Furthermore, by improving surgical outcomes, tension-reducing sutures establish a solid foundation for effective doctor-patient communication and postoperative guidance. Parents are more likely to accept and trust medical advice, follow instructions, and actively cooperate with healthcare providers, collectively contributing to higher parental satisfaction.

However, this study has certain limitations. First, its retrospective nature may introduce inherent biases. Second, the *post-hoc* power analysis revealed that the statistical power for comparing complication rates was only 55.2%, indicating that the study was underpowered to detect a statistically significant difference in this outcome, despite a clinically observed reduction. Therefore, the non-significant P-value for complications should be interpreted with caution, and larger prospective studies are needed to validate this finding. Third, the sample size included was relatively small, which may not fully reflect the effectiveness of tension-reduction sutures in different clinical conditions and among different pediatric patients, leading to potential sampling bias. Future research could further expand the sample size to include more children with various types of facial lacerations, thereby improving the reliability and generalizability of the findings. Fourth, the duration of follow-up in this study was relatively short. Scar formation and evolution is a long-term process, often taking 6–12 months or even longer to stabilize. At the 3-month postoperative mark, some scars may still be in the proliferative phase, characterized by redness and increased thickness. Over time, scars may gradually soften and fade in color. Therefore, the VSS score at 3 months may overestimate scar severity and fail to fully reflect the long-term inhibitory effect of tension-reduction sutures on scar maturation. Subsequent studies should extend the follow-up period to more comprehensively understand the long-term impact of tension-reduction sutures on scar formation. Finally, this study only compared tension-reduction sutures with conventional suturing methods and did not explore other potential treatment options. In the treatment of pediatric facial lacerations, other methods such as adhesive tape closure and biologic glue adhesion are also available. Future research could broaden the scope to compare the advantages and disadvantages of different treatment methods, providing more options and references for clinical practice.

## Conclusion

In summary, this study comprehensively evaluated the clinical value of tension-reduction sutures in the treatment of pediatric facial lacerations. The findings indicate that this suturing technique is more conducive to promoting primary wound healing and reducing scar formation. Additionally, it was associated with a numerical trend towards fewer postoperative complications and further enhance parental satisfaction with the surgical outcomes. These results provide certain guidance for the broader clinical application of this treatment method. Future studies with larger sample sizes and longer follow-up periods are warranted to confirm the long-term benefits of this technique.

## Data Availability

The raw data supporting the conclusions of this article will be made available by the authors, without undue reservation.
